# Sanshool improves UVB-induced skin photodamage by targeting JAK2/STAT3-dependent autophagy

**DOI:** 10.1038/s41419-018-1261-y

**Published:** 2019-01-08

**Authors:** Dan Hao, Xiang Wen, Lian Liu, Lian Wang, Xianli Zhou, Yanmei Li, Xin Zeng, Gu He, Xian Jiang

**Affiliations:** 1 Department of Dermatology and Venereology and State Key Laboratory of Biotherapy, West China Hospital, Sichuan University, Chengdu, 610041 P.R. China; 2 School of Life Science and Engineering, Southwest Jiaotong University, Chengdu, 610031 P.R. China

## Abstract

Ultraviolet radiation is markedly increased because of pollution and the depletion of the stratospheric ozone layer. Excessive exposure to sunlight can negatively affect the skin, resulting in sunburn, photo-aging, or skin cancer. In this study, we first determined the photoprotective effect of sanshool, a major component in *Zanthoxylum bungeanum*, on UVB-irradiated responses in human dermal fibroblasts (HDFs) and nude mouse. We found that sanshool treatment can protect cells against the effects of UVB irradiation by (i) increasing cell viability, (ii) inhibiting MMP expression, and (iii) inducing autophagy. We also used the recombinant CSF2 or anti-CSF2 antibody co-cultured with human dermal fibroblasts (HDFs) and found that CSF2 contributes to sanshool-induced autophagy. Sanshool hindered the UVB-induced activation of JAK2-STAT3 signaling in HDFs, thereby inhibiting the expression of MMPs and activation of autophagic flux. Exposure of the dorsal skin of hairless mice to UVB radiation and subsequent topical application of sanshool delayed the progression of skin inflammation, leading to autophagy and inhibiting the activation of JAK2-STAT3 signaling. These results provide a basis for the study of the photoprotective effect of sanshool and suggest that it can be potentially used as an agent against UVB-induced skin damage in humans.

## Introduction

The biological effects of UV radiation on skin are based on light absorption and intrinsic energy conversion^1^, which lead to facial wrinkling, pigmentation, roughness, and telangiectasias. Continuous exposure to UV radiation stimulates the synthesis of reactive oxygen species (ROS)^2^, promotes the expression of matrix metalloproteinase (MMP), and decreases procollagen synthesis^3^. MMPs are a family of structurally related matrix degrading enzymes, which play key roles in various destructive processes, including tumor invasion, inflammation, and skin aging. MMP-1 and MMP-3 are mainly related to the degradation of collagen, which is the major structural component in the extracellular matrix of the dermal connective tissue. Moreover, UV radiation can accelerate cellular aging associated with shortening of telomeres, oxidative stress, and genetic mutations and alter signal transduction pathways^4^. Long-term exposure to solar radiation not only induces actinic damage, such as sunburn and actinic dermatitis, but also contributes to the development of basal cell carcinoma and other skin cancers.

An increasing number of studies have recently focused on how plant extracts protect skin photodamage^5^. Sichuan peppers, also referred to as *Zanthoxylum bungeanum*, have been recognized for their medicinal and culinary properties in both traditional Asian and Native American cultures. A recent study demonstrated that *Z. bungeanum* is a functional cosmetic ingredient used to temporarily improve the appearance of skin wrinkles^6^. Hydroxy-α-sanshool (sanshool) is the main active ingredient in Sichuan peppers^7^. It has a conjugated long fatty chain, which allows it to absorb extra energy from UV radiation. On the basis of these characteristics, we hypothesized that sanshool protects the skin against photodamage.

In addition to triggering the DNA damage response signaling pathways, UV radiation induces autophagy, a catabolic process that clears unwanted or damaged proteins, lipids, and organelles. UV radiation of skin dermal fibroblasts can induce the formation of autophagosomes^8^, further protecting the cells to a certain level. Accordingly, the basal level of autophagy is reduced in aged cells^8^, which indicates that the upregulation of autophagy can be a potential intervention target for antiaging^9^.

CSF2, which is secreted by various cell types in response to cytokine or immune and inflammatory stimulation, was initially characterized as a growth factor; in addition, it stimulates the growth and differentiation of cells from various lineages^10^. Recent evidence indicates that endogenous autophagy is correlated with the release of CSF2 and subsequent activation of the JAK2-STAT3 and AKT pathways in radiation-induced breast cancer cell^11^.

This study was designed to verify the photoprotective effects of sanshool on ultraviolet B (UVB)-induced photodamage in normal human dermal fibroblasts (HDFs) in vitro and hairless mouse models in vivo. RNA sequencing, gene set enrichment analysis (GSEA), and experimental validation of bioinformatics results were performed to determine the mechanisms of sanshool on skin photoprotection. We found the involvement of GM-CSF, CSF2, and their associated genes, which provides a reliable direction for elucidating the molecular mechanisms of sanshool. The results of this study particularly show that the photoprotective effects of sanshool correlate with the activation of autophagy in vivo and in vitro, which is also attributed to the AKT-JAK2-STAT3 pathway. Our findings define the functional roles of sanshool in UVB radiation and provide a molecular basis for targeting JAK-STAT pathway-dependent autophagy to alleviate and treat photodamaged skin.

## Results

### Sanshool treatment protects HDFs against UVB irradiation

**Fig. 1:**
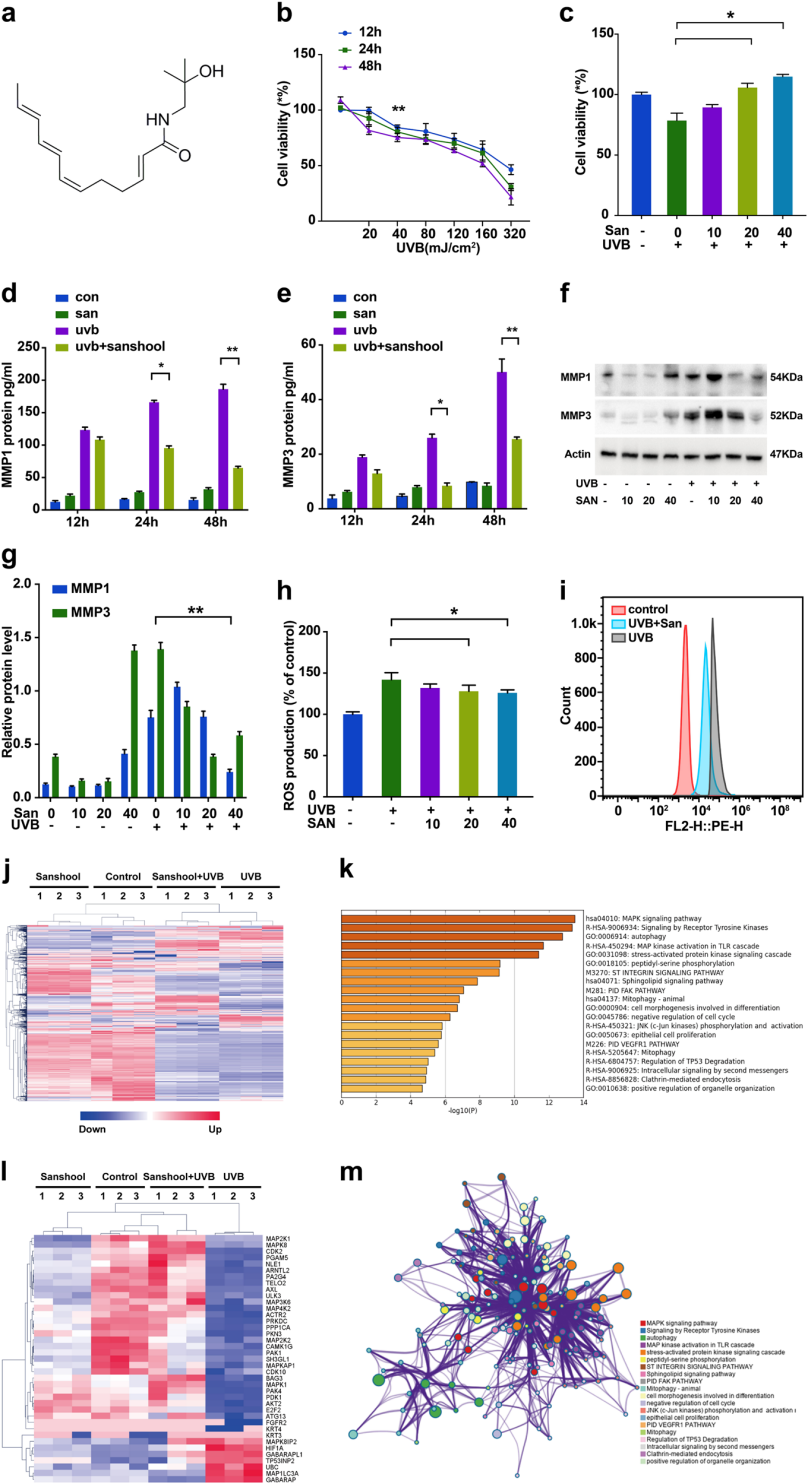
Protection of HDFs against UVB irradiation with sanshool treatment by improving cell viability and inhibiting MMP production. **a** Structure of sanshool; **b** HDFs irradiated with UVB at varying doses for 12, 24, and 48 h and effect of UVB irradiation on cell viability; **c** Effect of sanshool treatment on the cell viability of UVB-irradiated HDFs for 24 h. Cell viability measured by CCK-8 assay. Irradiated or non-irradiated HDFs at 50 mJ/cm, sanshool-treated at 40 μM for 12, 24, and 48 h; **d**, **e** Effect of sanshool treatment on the MMP-1 and MMP-3 secretion of UVB-irradiated HDFs, determined by ELISA; **f** Effects of sanshool treatment on the expression levels of MMP-1 and MMP-3 in 24 h; **g** Relative protein levels of MMP-1 and MMP-3; **h**, **i** Effects of sanshool treatment on ROS production; Data from representative experiments repeated 3 times with similar results. Results presented as means ± SD of three independent experiments (*n* = 3). **p < 0.005 compared with the UVB-treated group. UVB irradiation induced over 2700 differential expressed genes (DEGs), sanshool treatment of control HDFs induced about 960 DEGs, and 1170 DEGs were observed between the UVB group and the sanshool + UVB group. **j** Cluster of DEGs with UVB irradiation, sanshool treatment, or sanshool + UVB; **k** GO and KEGG enrichment analysis of DEGs between the UVB-irradiated group and the sanshool + UVB group; **l** Normalized autophagy-related DEGs in each group; **m** Enriched pathways and interactions of DEGs between the UVB-irradiated group and the sanshool + UVB group

We conducted GSEA on the reported microarray data set GSE41078 to identify the potential signaling pathways and biological processes involved in UVB-induced skin injury. The p53-hypoxia and IL1R pathways were identified as the two most significantly enriched pathways in GSEA (Fig. S1), and both pathways were related to inflammation. As one of the most important photoprotective compounds of *Z. bungeanum*, (Fig. 1a), sanshool was evaluated by assay for its capacity to protect HDFs against UVB-induced injury (Fig. 1b); a significant change was observed in cells exposed to UVB light >40 mJ/cm^2^. A further study was conducted on cells exposed to UVB; sanshool treatment dose-dependently improved the viability of UVB-irradiated cells in the range of 10–40 μM (Fig. 1c). This dose of sanshool was used in subsequent experiments and was found to substantially protect HDFs against UVB.

### Effects of sanshool on MMP expression and ROS generation in HDFs

UVB irradiation activates the secretion and expression of MMPs, which is the hallmark of skin aging^12^. We determined the MMP-1 and MMP-3 secretion of HDFs by ELISA under UVB irradiation with or without sanshool. The results indicate that sanshool treatment moderately increased the secretion of MMP-1 and MMP-3 secretion but significantly decreased the UVB-induced secretion of MMP-1 and MMP-3 (Figs. 1d, e). We also examined the expression of MMP-1 and MMP-3 by Western blot analysis (Figs. 1f, g), which revealed increases in UVB-induced HDFs. Meanwhile, sanshool (40 μM) significantly reduced the expression of MMP-1 and MMP-3 in HDFs treated with a UVB dose of 40 mJ/cm^2^.

ROS generation has been regarded as an initial event triggering UVB-mediated MMP expression^13^. Intracellular ROS accumulation is determined by DCF-DA probe with flow cytometry. In the current study, ROS levels increased under UVB irradiation (Fig. 1h). Sanshool treatment of HDFs reversed this effect, suggesting that sanshool inhibited ROS production in UVB-induced HDFs (Fig. 1i).

### mRNA sequencing of the effects of sanshool on UVB-irradiated response in HDFs

mRNA sequencing was performed on the following groups: UVB-irradiated HDFs, sanshool-treated HDFs, sanshool treated UVB-irradiated HDFs, and control HDFs. Figure 1j reveals the following: UVB irradiation induces more than 2700 differentially expressed genes (DEGSs); sanshool treatment of control HDFs induces about 960 DEGs; 1170 DEGs exist between the UVB group and the sanshool + UVB group. GO, KEGG, and Reactome enrichment analysis suggested that MAPK pathways and autophagy were most significantly enriched in the DEGs between the UVB group and the sanshool + UVB group (Fig. 1k). The relative expression levels of differentially expressed autophagy-related genes in each group are shown in Fig. 1l. The interaction network of the top 20 enriched pathways was visualized using Metascape (Fig. 1m).

### Sanshool treatment activates autophagic flux in UVB-irradiated HDFs

**Fig. 2:**
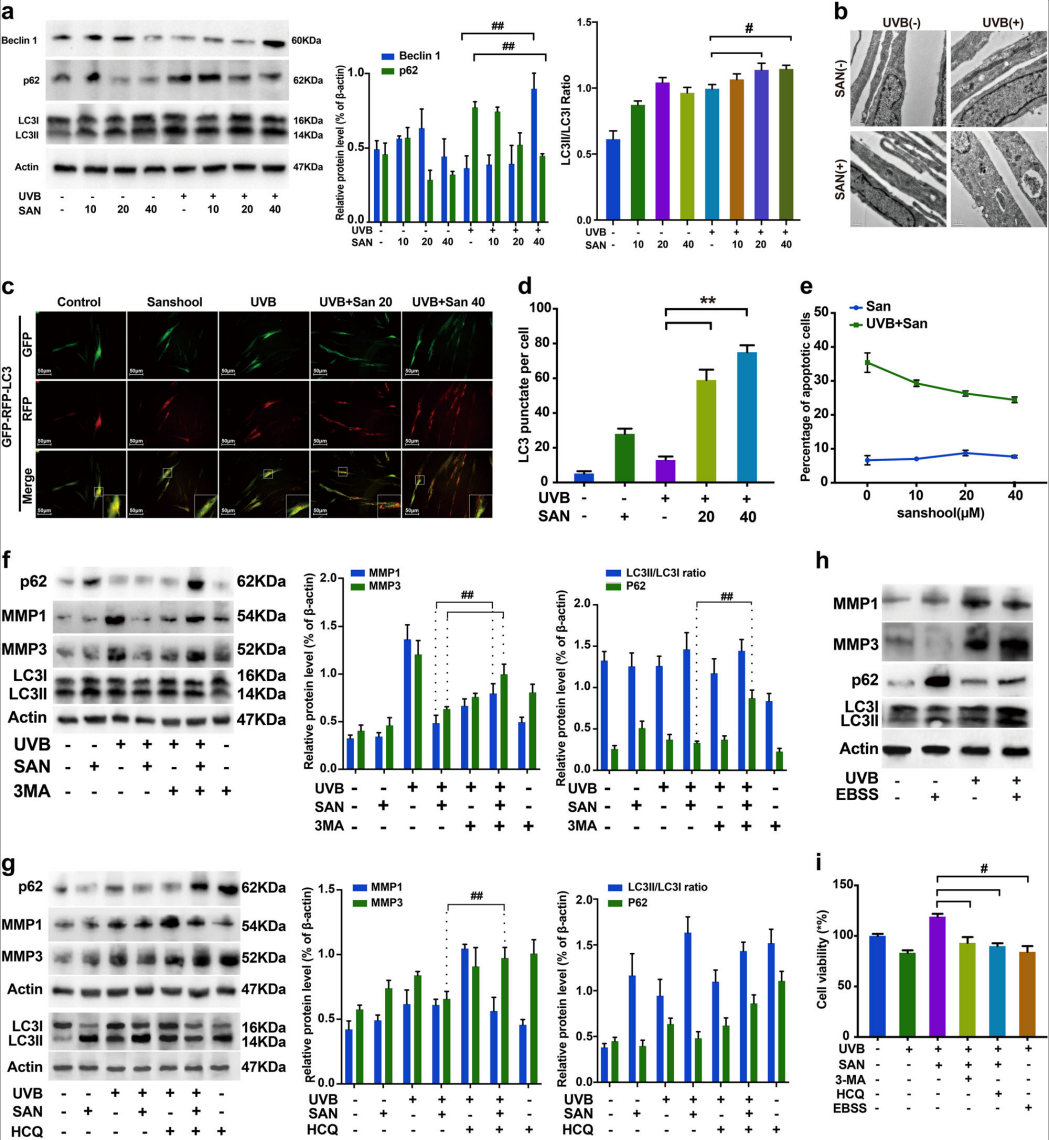
Activation of autophagic flux in UVB-irradiated HDFs with sanshool treatment. **a** UVB-irradiated HDFs or non-irradiated HDFs treated with sanshool; HDFs treated with sanshool (0, 10, 20, and 40 μM) for 24 h; Western blot analysis for measuring the expression levels of LC3I/II, Beclin1, p62, and β-actin. **b** Electron microscopy showing the ultrastructure of autophagosomes in sanshool-treated HDFs (40 μM); Arrows indicating autophagosomes, including residual digested material (black arrow). **c** Representative images of LC3 staining by measuring fluorescence intensity in HDFs in different groups infected with the RFP-GFP-LC3 adenovirus by fluorescence microscopy. **d** Quantification of punctate structures in adenovirus-GFP-RFP-LC3-positive HDFs treated with sanshool (20 and 40 μM) for 24 h. **e** Effects of sanshool treatment on apoptosis and necrosis in UVB-irradiated HDFs; UVB-irradiated HDFs treated with sanshool (0, 10, 20, and 40 μM) for 24 h, and apoptosis measured by Annexin V/PI dual staining, followed by flow cytometry. **f**, **g** UVB-irradiated or non-irradiated HDFs pre-incubated with 3-MA (10 mM) and HCQ (30 μM) for 6 h, followed by sanshool treatment for 24 h; Western blot analysis for measuring the expression levels of LC3I/II, MMP-1, MMP-3, p62, and actin. **h** HDFs treated with EBSS after UVB irradiation; Expression levels of LC3I/II, MMP-1, MMP-3, p62, and actin measured by Western blot analysis. **i** Viability of cells treated with sanshool, 3-MA, HCQ, EBSS, and/or UVB irradiation. Results presented as means ± SD of three independent experiments (*n* = 3). *Comparison with UVB-treated group, *P* < 0.05

Autophagy is responsible for the clearance of damaged proteins and organelles. The presence of aging-associated MMP-1 and MMP-3 prompted us to investigate whether autophagic flux could be detected. We used several methods to determine whether sanshool treatment induces autophagy in UVB-irradiated HDFs. LC3II expression is proportional to the number of autophagic vacuoles. We found that sanshool significantly increased LCII/LC3I in UVB-irradiated HDFs, although UVB irradiation, solely, could improve the autophagy level. We also measured the expression levels of Beclin1 and p62, which were proved to be key proteins during autophagosome formation^14^; beclin1 expression increased in the sanshool-treated cells (Fig. 2a). The p62 expression of UVB-irradiated HDFs decreased and the expression of Beclin 1 increased in the sanshool-treated UVB-irradiated HDFs. Electron microscopy analysis indicated the presence of more autophagosome-like vacuoles in the cytoplasm of the sanshool-treated HDFs (Fig. 2b). In addition, we used the tandem GFP-RFP-LC3 adenovirus construct to verify autophagy induction by the formation of punctate structures, representing autophagosome formation. The rationale for this assay is based on the difference in pH between the acidic autolysosomes and the neutral autophagosomes, as well as the difference in pH sensitivity between GFP and RFP to monitor progression from autophagic flux^15^. After the GFP-RFP-LC3 adenovirus was successfully introduced, LC3 and more punctate structures accumulated in the sanshool-treated UVB-irradiated HDFs (Fig. 2c). The quantitation of LC3-positive punctate structures per cell is shown in Fig. 2d. Moreover, the extent of apoptosis was quantified by flow cytometry analysis of sanshool-treated and control cells exposed to 40 mJ/cm^2^ UVB. UVB irradiation of cells resulted in 32.7% apoptotic cells (Fig. 2e). Sanshool treatment prevented cells from UVB-irradiated apoptosis in a dose-dependent manner. We further treated HDFs with autophagy inhibitor 3-methyladenine (3-MA)^16^ and hydroxychloroquine (HCQ)^17^. No blocking effects of autophagy production were observed after sanshool treatments in the 3-MA and HCQ pre-cultured groups. No significant difference in the LCII/LC3I ratio was found between groups (Fig. 2f, g). The p62 expression was increased by both 3-MA and HCQ. Notably, the expression levels of MMP-1 and MMP-3 in the cells pre-cultured with 3-MA, as well as MMP-3 in the cells pre-cultured with HCQ were increased relative to those of the cells simply treated with sanshool in UVB-irradiated HDFs. GFP-RFP-LC3 adenovirus transfection was conducted to confirm the formation of punctate autophagosomes. The influence of 3-MA and HCQ on the autophagosome structures in HDFs with a time-dependent manner. HCQ is typically an autophagy flux inhibitor. In agreement with some recently reports^18^, the autophagosome accumulation by HCQ treatment is a dynamic process, which usually sustained several hours after HCQ incubation. And the HCQ treatment could impair the autophagosome recurring with the same stimulus. Earle’s balanced salt solution (EBSS), a saline solution with physiological pH, was used to induce autophagy^19^. Cell death was observed in the EBSS-treated cells, which were considered as the control (Fig. 2i). The production of two classic aging markers, MMP-1 and MMP-3, were not inhibited by EBSS (Fig. 2h).

### CSF2 contributes to sanshool-induced autophagy in HDFs

**Fig. 3:**
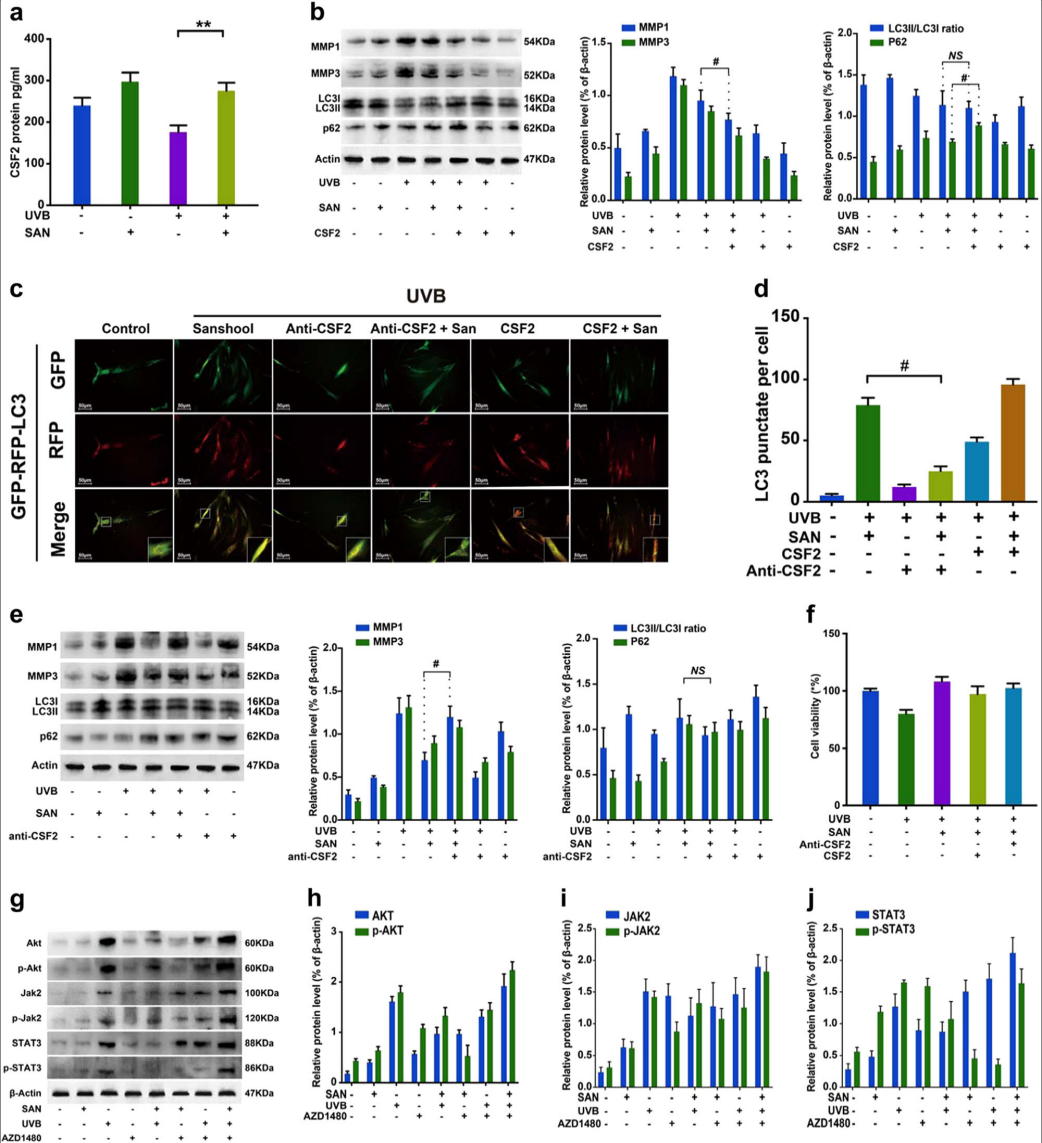
Sanshool treatment induces autophagy and the CSF2-dependent JAK2–STAT3 signaling pathway in UVB-irradiated HDFs. **a** HDFs treated with sanshool for 24 h after UVB irradiation; analysis of CSF2 secretion by ELISA; UVB-irradiated HDF or non-irradiated HDFs treated with sanshool when co-cultured with CSF2 (0.1 ng/mL). **b** Expression levels of LC3I/II, p62, MMP-1, MMP-3, and β-actin measured by Western blot analysis. **c** Representative images of LC3 staining by measuring fluorescence intensity in HDFs in different groups of cells infected with the RFP-GFP-LC3 adenovirus for 24 h by fluorescence microscopy. **d** Quantitation of punctate structures per cell, based on the number of punctate structures in GFP-RFP-LC3-positive cells. Addition of a CSF2-neutralizing antibody (5 μg/mL) to the medium in each group and incubation with sanshool in UVB-irradiated HDFs; **e** Expression levels of LC3I/II, p62, MMP-1, MMP-3, and actin measured by Western blot analysis; **f** Cell viability in response to CSF2 depletion or CSF2 oversupply. **g** Expression levels of JAK2, phospho-JAK2, STAT3, phospho-STAT3, AKT, and phospho-AKT after sanshool and/or AZD1480 treatment of UVB-irradiated or non-irradiated HDFs for 24 h, determined by Western blot analysis. (**h**) Relative protein expression levels of AKT and phospho-AKT. **i** Relative protein expression levels of JAK2 and phospho-JAK2; **j** Relative protein expression levels of STAT3 and phospho-STAT3. Results presented as means ± SD of three independent experiments (*n* = 3). *Compared with the UVB-treated group, *P* < 0.05

A recent study demonstrated that CSF2 is secreted by senescent fibroblasts^20^. In addition, rhGM-CSF repairs wounds in epithelial cells and keratinocytes^21^. We found that CSF2 could largely contribute to the effects of sanshool on UVB-irradiated HDFs. ELISA analysis indicated that CSF2 concentrations in the sanshool-treated UVB-irradiated HDFs were significantly increased relative to those in the UVB-irradiated HDFs (Fig. 3a). The addition of sanshool could promote the secretion of CSF2 regardless of whether the HDFs were subjected to UVB irradiation. Moreover, rhCSF2 was used to evaluate whether CSF2 affected the treatment of sanshool on HDFs via the regulation of cell viability and autophagy. With CSF2 addition, LC3-II conversion was accelerated, and p62 protein accumulated in UVB-irradiated HDFs (Fig. 3b). The number of LC3-positive punctate structures was increased in the presence of CSF2 relative to that in the UVB + sanshool group but was significantly decreased in the anti-CSF2 + UVB + sanshool group (Figs. 3c, d). The result was in accordance with the changes in MMP and autophagic marker. The expression and secretion of MMP-1 and MMP-3 were also decreased in the presence of CSF2 (Fig. 3b). Meanwhile, CSF2 neutralization decreased the LC3-II expression and largely promoted MMP-1 and MMP-3 expression, alleviating the protective effects of sanshool on UVB-irradiated HDFs (Fig. 3e). CSF2 neutralization slightly inhibited the protective effect of sanshool on cell viability. Notably, the presence of CSF2 exerted no positive effects on cell viability (Fig. 3f).

### Sanshool protects HDFs from UVB irradiation by inhibiting JAK2-STAT3 pathway activation

**Fig. 4:**
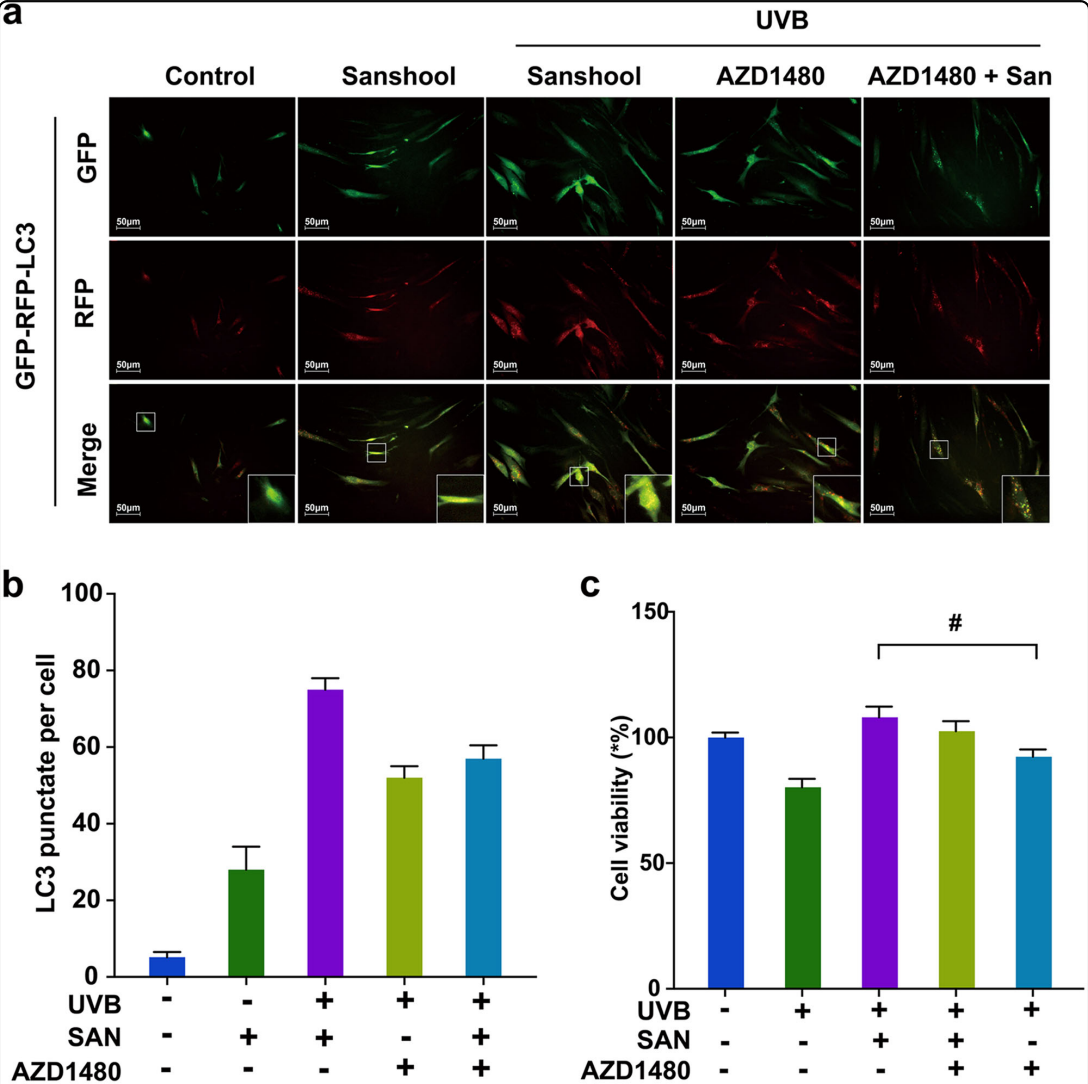

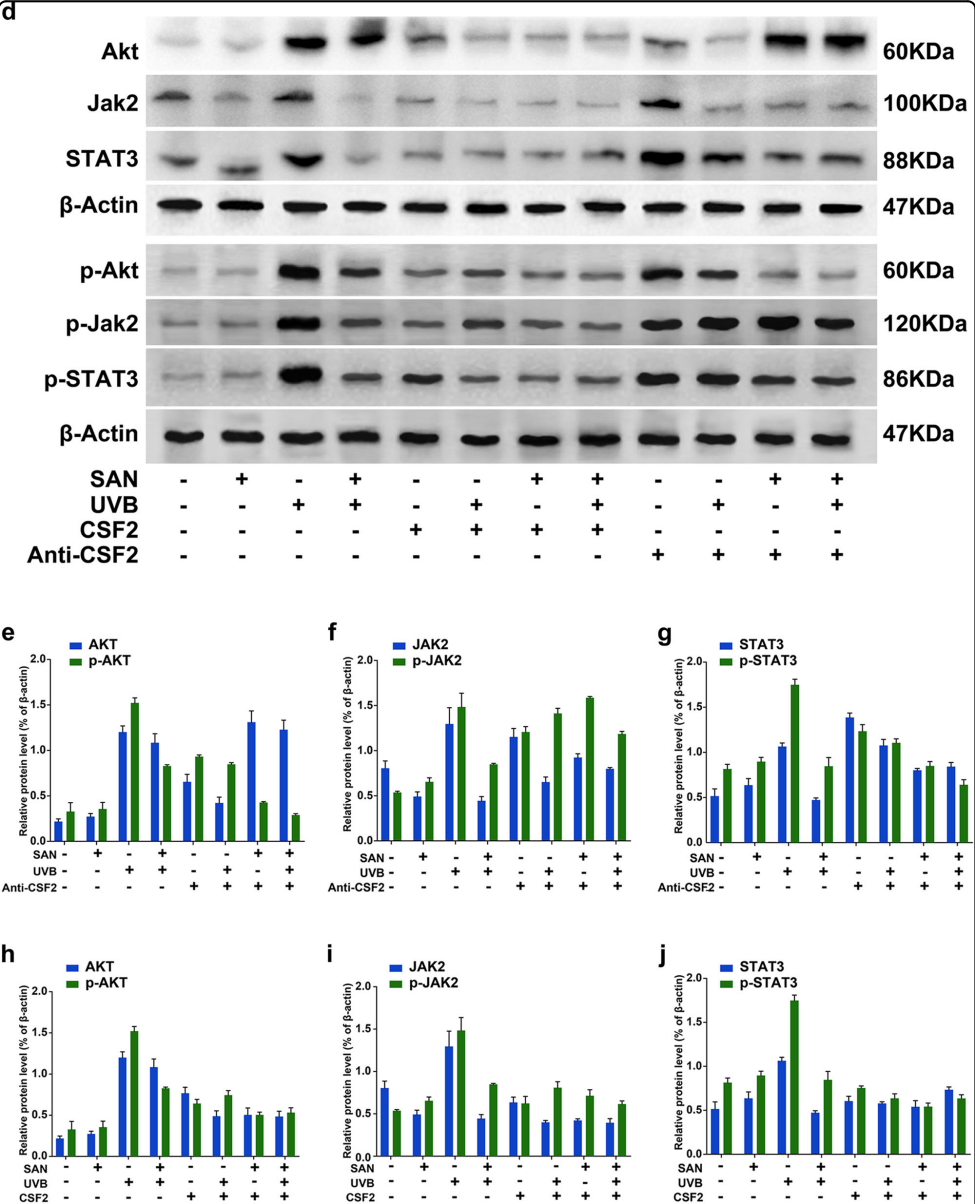
Sanshool protects HDFs from UVB irradiation by inhibiting the AKT–JAK2–STAT3 pathway activation. **a** Representative images of LC3 staining by measuring fluorescence intensity of HDFs in different groups of cells infected with RFP-GFP-LC3 adenovirus for 24 h by fluorescence microscopy. **b** Quantitation of punctate structures per cell, based on the number of punctate structures in GFP-RFP-LC3-positive cells. **c** Cell viability in response to AZD1480. **d** Expression levels of JAK2, phospho-JAK2, STAT3, phospho-STAT3, AKT, and phospho-AKT measured by Western blot analysis in response to CSF2 depletion or CSF2 oversupply. **e**, **h** Relative protein expression levels of AKT and phospho-AKT in response to CSF2 depletion and CSF2 oversupply. **f**, **i** Relative protein expression levels of JAK2 and phospho-JAK2 in response to CSF2 depletion and CSF2 oversupply. **g**, **j** Relative protein expression levels of STAT3 and phospho-STAT3 in response to CSF2 depletion and CSF2 oversupply. Results presented as means ± SD of three independent experiments (*n* = 3). *Compared with the UVB-treated group, *P* < 0.05

Figure 3g shows that the expression and phosphorylation levels of AKT (Fig. 3h), JAK2 (Fig. 3i), and STAT3 (Fig. 3j) are significantly upregulated in UVB-irradiated HDFs, as determined by Western blot analysis. Meanwhile, sanshool treatment inhibited the expression and phosphorylation of JAK2, STAT3, and AKT in UVB-irradiated HDFs. We administered a JAK2 inhibitor (AZD1480), which significantly decreased p-STAT3 and p-AKT expression (Fig. 3h, j). Confirmation of the autophagic level by GFP-RFP-LC3 adenovirus transfection indicated that pre-treatment with AZD1480 could improve the sanshool-induced autophagy level (Fig. 4a, b). The treatment with sanshool alleviated the inhibitory effects of AZD1480 on UVB-irradiated cells (Fig. 4c). These data indicate that JAK2 inhibition failed to intensify the JAK2-STAT3 pathway by sanshool treatment.

In addition, the JAK2-STAT3 signaling pathway is associated with a mass of growth factors, cytokines, and hormones^22^. We also examined whether CSF2 could mediate the AKT-JAK2-STAT3 pathway in sanshool-treated HDFs (Fig. 4d). The cells were pre-cultured HDFs with CSF2 or a CSF2-neutralizing antibody. Pre-culture treatment with the CSF2-neutralizing antibody exerted no effect on the expression levels of AKT in each group (Fig. 4e), however the expression of p-AKT also decreased in the sanshool treatment groups. The expression levels of p-JAK (Fig. 4f) and p-STAT3 (Fig. 4g) significantly increased in each group when pre-cultured with CSF2-neutralizing antibody (compared with UVB + sanshool group). Pre-culture treatment with CSF2 could decrease the expression of AKT in UVB-irradiated HDFs (Fig. 4h). CSF2 combined with sanshool could decrease the phosphorylation and expression levels of JAK2 (Fig. 4i) and STAT3 (Fig. 4j) in UVB-irradiated HDFs. These results suggest the involvement of CSF2 in the JAK2-STAT3 pathway target for sanshool in HDFs. The interaction between AKT and CSF2 was reduced. CSF2 probably participated in activating the JAK2-STAT3 pathway in sanshool-treated HDFs.

### Sanshool alleviates UVB-induced skin photodamage in mouse skin

**Fig. 5:**
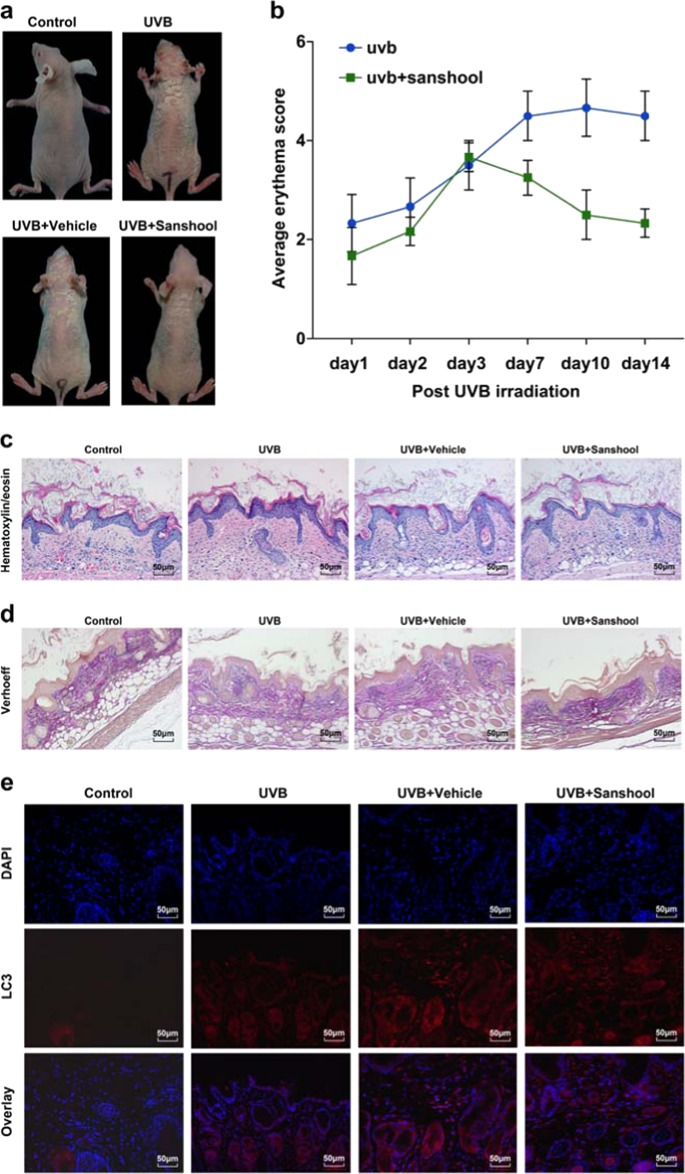

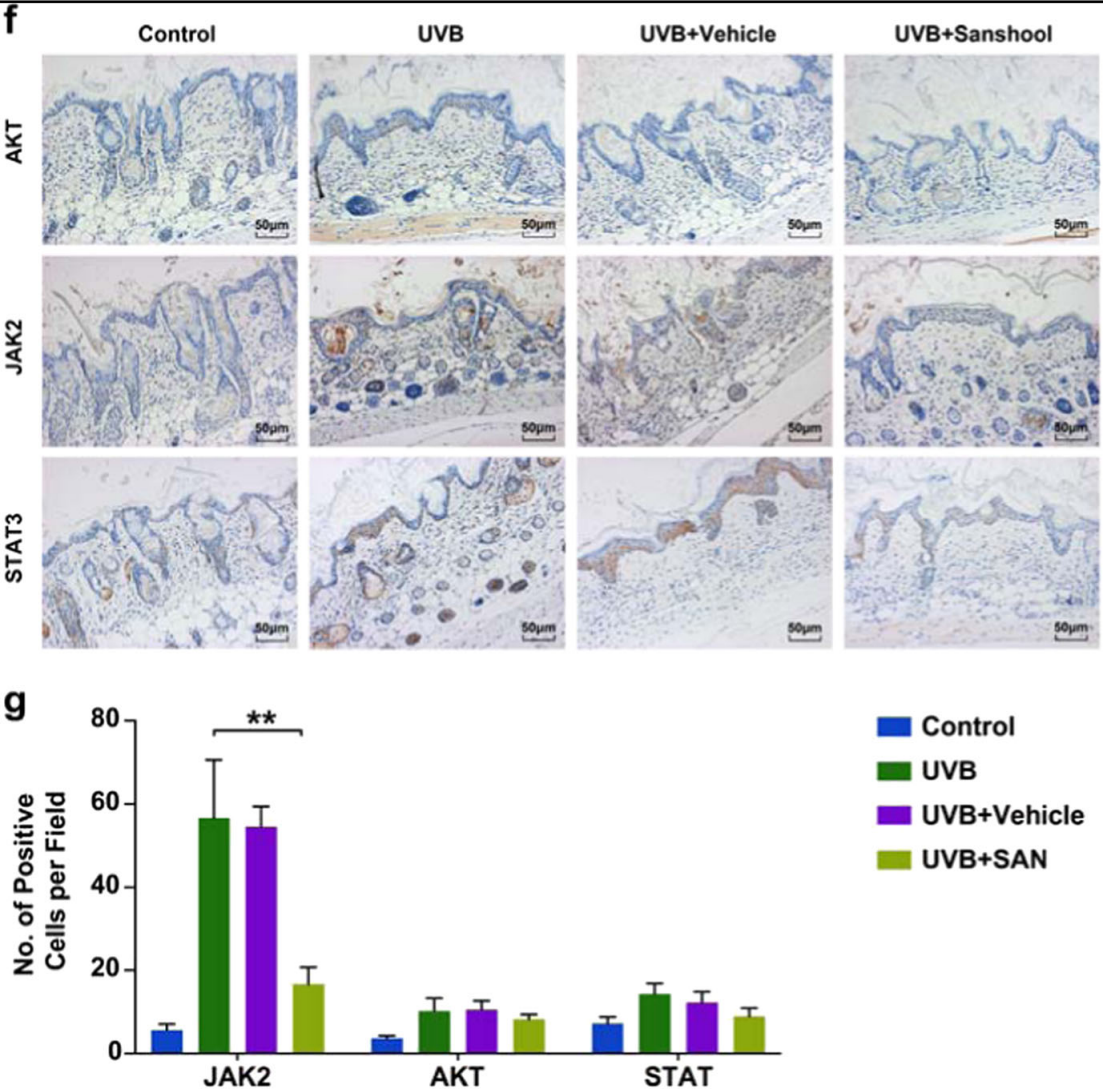
Sanshool alleviates UVB-induced skin photodamage in mouse skin. Topical application of sanshool (20 mg/kg) immediately after UVB irradiation. The groups of mice were either (i) unexposed (control), (ii) treated topically with sanshool (20 mg/kg) on the dorsal skin, (iii) exposed to UVB radiation (300 mJ/cm^2^), or (iii) treated topically with sanshool (20 mg/kg) on the dorsal skin thrice a week for 2 weeks, as described under Materials and methods. After 2 weeks, the animals were sacrificed, and skin biopsies were processed for staining. Induction of erythema and scales on the dorsal skin of hairless mice treated with sanshool and/or UVB irradiation. **a** Representative features of the dorsal skin 2 weeks after UVB irradiation in two mice from each group; Measurement of average erythema score of the dorsal skin of hairless mice without UVB irradiation or average erythema score of the dorsal skin of hairless mice 1, 2, 3, 7, and 14 after UVB irradiation. Values presented as mean ± SD. H&E (**c**) staining and Verhoeff’ elastic stain (**d**) of skin samples (scale bar = 100 μm). **e** Immunofluorescence staining using an active LC3 antibody (**e**, scale bar = 50 μm) and **f** immunohistochemical staining for JAK2, STAT3, and AKT. **g** Quantitation of positive stained cells per field for JAK2, STAT3, and AKT. Results presented as means ± SD of three independent experiments (*n* = 3). *Compared with the UVB-treated group, *P* < 0.05

The protective effects of sanshool in UVB-irradiated HDFs via autophagy induction and AKT-JAK2-STAT3 pathway inhibition prompted us to directly test whether sanshool could immediately rescue mouse skin after UVB injury in the same manner. Sanshool was topically applied to the dorsal trunk of nude mice, followed by UVB irradiation once daily for 2 weeks. Macroscopic photos showed that the UVB-irradiated skin was considerably drier and paler than the non-irradiated skin. Increased scales were observed in UVB radiation-induced mice versus vehicle-treated mice. These superficial changes were markedly reduced with sanshool treatment (Fig. 5a).

First, the erythema on the dorsal skin of mice peaked at 72 h after UVB irradiation in all groups, with the sanshool-treated mice showing less severe erythema and less scales compared with the vehicle-treated group and untreated group (Fig. 5a). A significant difference between the sanshool-treated group and the vehicle group was observed after UVB irradiation (Fig. 5b). Skin histological analysis by hematoxylin and eosin (H&E) staining indicated that UVB irradiation led to more inflammatory responses such as increased thickness, epidermal hyperplasia with enlarged sebaceous glands, and hyperkeratosis in the skin of mice, compared with the vehicle-treated mice. Meanwhile, topical application of sanshool reduced these morphological changes, indicating the photoprotective activity of sanshool in the skin (Fig. 5c). Verhoeff–van Gieson elastic staining is a simple method used to visualize elastic fibers. Verhoeff staining showed an increase in black-stained elastic fibers and a decrease in the density of pink-stained collagen in UVB-irradiated skin (Fig. 5d). The elastic fibers and collagen density of mice topically applied with sanshool increased. Immunofluorescence staining and corresponding quantification showed differences in the endogenous LC3 levels as well as the number and overall distribution of LC3-positive punctate structures between the vehicle-treated and the sanshool-treated, UVB-irradiated mice (Fig. 5e).

We then determined the expression levels of AKT, JAK2, and STAT3 in each group by immunohistochemistry (Fig. 5f). Consistent with the in vitro results, JAK2 increased in the UVB-irradiated group (Fig. 5g). Meanwhile, the activation of JAK2 was effectively suppressed by the topical application of sanshool. These results were generally consistent with those of the UVB-irradiated HDFs and suggested that sanshool exhibited skin-protective activities against UVB irradiation.

## Discussion

Repeated and chronic exposure of skin to solar radiation induces various skin damage, such as actinic dermatitis and skin cancers. Photoprotective agents and measures aimed at reducing sun exposure and preventing the development of acute and chronic actinic damage are highly recommended by dermatologists. Psychophysical studies have identified sanshool as the compound responsible for the distinct tingling and buzzing sensations produced by Sichuan peppers^23^. Another study demonstrated that extraction of Sichuan pepper also exerts a lifting effect, showing visible and transient improvement of facial wrinkles^6^. These results prompted us to explore the potential of sanshool in preventing UVB-induced skin damage.

In the present study, the photoprotective effect of sanshool on skin was first evaluated as a potential therapeutic agent for photodamaged skin. MMP-1 and MMP-3 are mainly associated with the degradation of collagen, which is the major structural component in the extracellular matrix of the dermal connective tissue^24^. Meanwhile, the upregulation of MMP-1 and MMP-3 can be inhibited by sanshool treatment. These results demonstrated that sanshool could alleviate UVB-induced fibroblast photodamage by suppressing MMP production. Autophagy is a catabolic process in which damaged organelles and proteins are engulfed and degraded to provide metabolic needs. An important aspect of our study was to determine whether autophagy induction was related to sanshool-based therapeutic intervention. We found that sanshool treatment of UVB-irradiated HDFs induced autophagy. Moreover, the increase in autophagy failed to promote cell apoptosis, suggesting that sanshool could balance autophagy and cell apoptosis, as well as protect UVB-irradiated cells from programmed death. In the present study, sanshool-induced autophagy was blocked using the inhibitors 3-MA and HCQ. Notably, the expression levels of MMP-1 and MMP-3 were elevated after pre-treatment with both 3-MA and HCQ. This increase suggested that autophagy inhibition could worsen photodamage in fibroblasts. In addition, topical application in vivo of sanshool on the dorsal skin of UVB-irradiated mice similarly induced LC3-II expression, strongly suggesting that sanshool exerted a specific autophagy-inducing effect.

A previous study demonstrated that X-ray-irradiated senescent cancer cells could exert bystander effects, which could be mediated by autophagy in breast cancer cells by releasing CSF2 and activating the JAK2-STAT3 and AKT pathways^11^. Our RNA-Seq analysis identified multiple genes related to sanshool treatment and UVB irradiation in which the most remarkable results referred to CSF2 and the AKT-JAK2-STAT3 pathway. The JAK2-STAT3 signaling pathway, a core component of organ protection, is involved in various organs^25^. In the current study, sanshool exerted positive effects on the expression and secretion of CSF2 in UVB-irradiated HDFs. The autophagy level was elevated by the addition of CSF2 and was consistent with the reductions in the MMP-1 and MMP-3 expression levels of UVB-irradiated HDFs. Moreover, we found that sanshool protected HDFs from UVB irradiation by inhibiting the JAK2/STAT3 pathway via CSF2 mediation. Our study was the first to indicate that UVB irradiation activates the JAK2-STAT3 pathway by increasing the expression levels of JAK2 and STAT3. Sanshool treatment could inhibit the JAK2-STAT3 pathway. Moreover, AZD1480, an ATP-competitive small molecule inhibitor of JAK2, was used to attenuate the JAK2/STAT3 signaling pathway, which could enhance the protective capacity of sanshool by intensifying autophagy and improving cell viability. AKT expression, which was shown to affect the activation of autophagy flux, presented changes similar to those of JAK2-STAT3 in the present study. These results indicated that the photoprotective effects of sanshool depended on the inhibition of the JAK2/STAT3 pathway.

In summary, the results of our study suggested that sanshool treatment of HDFs and mouse skin promoted autophagy flux, which was mediated by CSF2 via the inhibition of the AKT and JAK2-STAT3 pathways, thereby protecting cells from UVB-induced photodamage. Therefore, these results indicated that sanshool could potentially protect against the adverse effects of UVB radiation via the modulation of autophagy and inhibition of MMP expression. These results provide a basis for the photoprotective effect of sanshool and suggest its application as an agent against UVB-induced damage to human skin. Our findings define the functional roles of sanshool in UVB irradiation and provide molecular basis for targeting JAK-STAT pathway-dependent autophagy to alleviate and treat photodamaged skin.

## Materials and methods

### Cell culture and UVB radiation

Normal HDFs were obtained from National Infrastructure of Cell Line Resource (China) and maintained in Dulbecco’s Modified Eagle’s Medium containing 10% fetal bovine serum and 1% penicillin–streptomycin (Life Technologies) in 5% CO_2_ at 37 °C. Prior to UV irradiation, the cells were washed and covered with a thin layer of PBS. Mock-irradiated controls followed the same schedule of medium changes without UVB irradiation. For irradiation, a UVB lamp (SIGMA, China) was used, which delivered uniform irradiation at a distance of 15 cm. Radiation intensity was monitored using a UVB light meter. mRNA sequencing was performed on three controls and three UVB-irradiated HDF samples on an Illumina HiSeq4000 platform in accordance with the Illumina Tru-Seq protocol. The sequenced reads were aligned against the *Homo sapiens* genome by using the Human hg38 transcript set. The transcript-level counts were imported and analyzed for differential expression by using the software DESeq2.

### ELISA and Western blot assays

Secretion of MMP-1(P03956), MMP-3 (P08254, Raybiotech, Inc.), and CSF2 (DGM00, R&D Systems) was detected using an ELISA kit in accordance with the instruction provided by the manufacturer. Proteins were extracted using a radioimmunoprecipitation assay buffer (Beyotime Institute of Biotechnology). Proteins were quantified and separated by 8–10% SDS-PAGE. After electrophoresis, proteins were transferred onto polyvinylidene fluoride membranes (Millipore). The membranes were blocked with nonfat milk for 2 h. The membranes were then incubated using primary antibodies overnight at 4 °C and incubated with HRP-conjugated secondary antibodies (Beyotime Institute of Biotechnology) for 1 h at room temperature. The blots were visualized using an enhanced chemiluminescence reagent (Beyotime Institute of Biotechnology). The band densities were analyzed using the software ImageQuant (Bio-Rad, ChemiDoc MP).

### Animal study

All animal experiments were reviewed and approved by the Institutional Animal Care and Use Committee of the West China Hospital. Female nude mice aged 6 weeks were provided by the Animal Center of Sichuan University (China). Mouse dorsal skin (each group, *n* = 4) was exposed to UVB irradiation at 300 mJ/cm^2^. This irradiation energy was selected based on recently published studies^26^. Samples were applied topically with a vehicle [1,2-dihydroxybutane-butyleneglycol] or 20 mg/kg sanshool following UVB irradiation 3 times a week for 2 weeks. Sanshool concentration was fixed at ~20 μM in accordance with the results of the study in vitro.

### Evaluation of average erythema score

After UVB irradiation, erythema in the skin of mice from each group was evaluated using a previously described method^26^. Mean grades were calculated for all mice at each time point after UVB irradiation.

## Supplementary information

suppl mater
