# Supplementary material for: Sanshool improves UVB-induced skin photodamage by targeting JAK2/STAT3-dependent autophagy
Source: Cell Death Dis. 2019 Jan 8;10(1):19. doi: 10.1038/s41419-018-1261-y (PMC6325150; doi:10.1038/s41419-018-1261-y)
Supplement: Supplementary file 1 — suppl mater [file 41419_2018_1261_MOESM1_ESM.docx]

**Supplement materials**

**
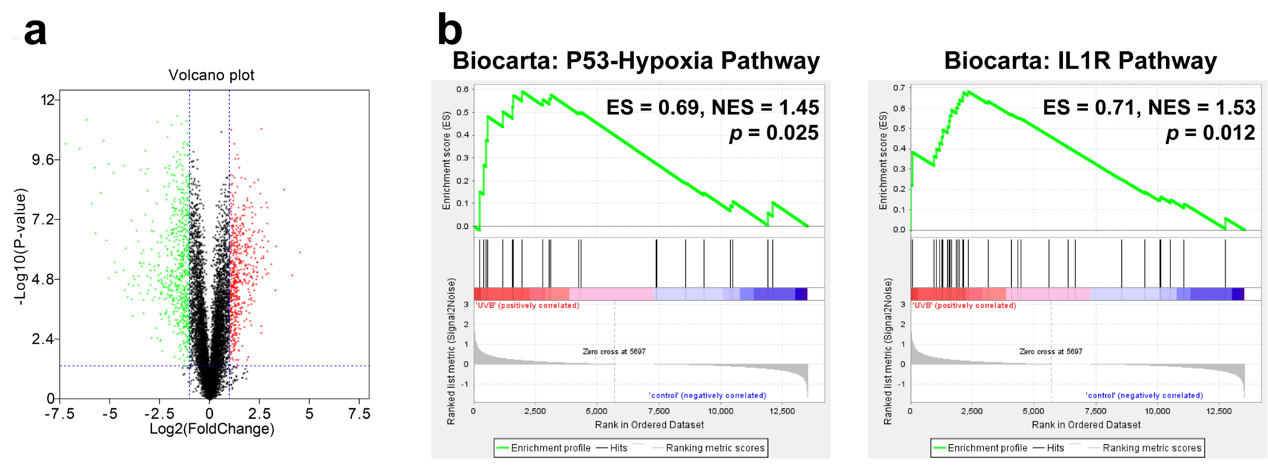
**

Figure S1. (a). Volcano plot of DEGs in UV-irradiated s kin tissues from GSE41078; (b) GSEA plot showing the enrichment of the p53-hypoxia and IL1R signaling pathways in UV-irradiated skin tissues.

**Materials and methods**

Reagents and antibodies

3-Methyladenine (3-MA; M9281) was purchased from Sigma-Aldrich Chemical. HCQ and AZD1480 (S2162) were supplied by Selleck Chemicals. MAP1LC3B (2775; 1:1000), SQSTM1 (5114; 1:1000), anti-beclin1 (3495; 1:1000), phospho–JAK2 (Tyr1007/1008, 3771; 1:1000), JAK2 (3230; 1:1000), phospho-STAT3 (Tyr705, 9131; 1:1000), STAT3 (9132; 1:1000), phospho–AKT (Ser473; 9271; 1:1000), and AKT (9272; 1:1000) antibodies were supplied by Cell Signaling Technology. Antibodies against human MMP-1 (MAB901; 1:1000) and MMP-3 (MAB548; 1:1000) were obtained from R&D Systems. Horseradish peroxidase (HRP)-conjugated antirabbit and antimouse antibody were provided by ZSBiO (China). All other chemicals used were of analytical grade and supplied by from Sigma-Aldrich (China).

**Plant Material**

The fruits of *Z. bungeanum* were collected in Hanyuan, Sichuan Province, China in Aug 2014. A voucher specimen (No. Z36151102) is deposited in School of Life Science and Engineering, Southwest Jiaotong University, Sichuan, China.

**Isolation and Identification of The mixture of Sanshools**

The pericarps of *Z. bungeanum* (12 kg) were extracted with EtOAc at room temperature to obtain the extract (800 g). The EtOAc extract (750 g) was subjected to a silica gel column eluted in a step gradient manner with petroleum ether–EtOAc (5:1 - 0:1) to afford fractions (A-C) based on TLC analysis. Fraction B (350 g) was subjected to silica gel column, eluted with petroleum ether: EtOAc (4:1) to Fractions B_1_-B_3_. Fractions B_2_ (105 g), which was future was subjected to CC over silica gel and eluted with petroleum ether–CH_2_Cl_2_ (50:1 - 1:0) to get hydroxy Sanshools (50 g), purity > 95% (HPLC).

Microarray data processing and visualization

The microarray dataset GSE41078 was downloaded from the Gene Expression Omnibus which contains 10 paired UVB-injured and adjacent normal skin tissues samples. Profile data extractions were performed based on the R and Bioconductor environment. GSEA was performed using GSEA 2.2.1 (http://www.broadinstitute.org/gsea).

Cell viability

The effect of sanshool or UVB (or both) on cell viability was determined by CCK-8 assay (Dojindo). After incubation for specified times at 37 °C in a humidified incubator, the cells were washed and exposed to UVB (10–150 mJ/cm^2^). The cells were plated at 1*10^4^ cells per well in 200 mL of complete culture medium containing sanshool in 96-well plates for 12, 24, and 48 h. Each concentration of sanshool was repeated in 6 wells. CCK-8 was added to each well and then incubated for 1 h. Absorbance was recorded on a microplate reader at 450 nm wavelength.

Cell apoptosis and necrosis assay

HDFs (1.5*10^6^ cells/mL) were seeded into 12-well plates and subjected to various treatments as previously described. Detection of apoptosis by flow cytometry was performed using the Annexin V/Propidium Iodide (PI) Apoptosis Detection Kit (Keygen Biotech, Nanjing, China). Staining was performed in accordance with instructions provided by the manufacturer. Cells were washed 2 times with PBS and then resuspended in a binding buffer. The solution was transferred into 1.5 mL culture tubes and then 5 μL Annexin V-FITC and PI was added. After incubation for 15 min at room temperature under dark conditions, the apoptotic rates were analyzed by flow cytometry (Beckman CytoFLEX, USA).

Measurement of ROS production

UVB-irradiated cells were incubated with sanshool for 12 h in serum-free medium, and ROS production was measured after staining with DCF–DA (Beyotime. China) for 15 min by flow cytometry (Beckman CytoFLEX, USA) in accordance with the instructions provided by the manufacturer.

Transmission electron microscopy

Cells were initially fixed with 2% glutaraldehyde in a 0.1 M sodium cacodylate–HCl buffer with 0.1 M sucrose (pH 7.4) for 2 h at 4 °C and then post-fixed with 1% OsO4 in a 0.15 M sodium cacodylate–HCl buffer for 1 h. The cells were gradually dehydrated in ethanol and embedded in Epon 812. Ultrathin sections were cut on a Reichert Ultracut S Ultramicrotome, mounted on copper grids, air-dried, and further stained with uranyl acetate and lead citrate. Sections were examined and then photographed with an electron microscope at 100 kV.

Adenoviral transduction

Cells were plated in 6-well plates (10^5^/well) in DMEM with 10% FBS one day before transfection. The cells were washed with PBS twice and then transfected with 20 μL of GFP-mRFP-LC3 adenovirus (MOI=100) (Hanbio Biotechnology, Shanghai, China)), which had been diluted in 2 mL DMEM (without FBS). The cells were incubated in the dark. After 6 h, the transfection medium was removed and replaced with fresh DMEM with 10% FBS. The cells were then cultured for 12 h for the subsequent treatment (UVB irradiation or/and sanshool). After the treatment, the cells were fixed with 1% PFA in PBS for 15 min at room temperature in the dark, thoroughly washed with PBS, and stained with DAPI for viewing under a fluorescence microscope(Zeiss, OBSERVER D1/AX10 cam HRC, China). The number of GFP and RFP dots was determined by manually counting the fluorescent punctate structures from at least 4 different fibroblast preparations with a 60× objective. The number of dots/cell was calculated by dividing the total number of dots by the number of nuclei in each microscopic field.

Histological analysis

Samples were fixed in 4% paraformaldehyde in PBS for 24 h, washed with tap water, dehydrated with graded ethanols, and embedded in paraffin wax. Blocks of paraffin wax were cut in 4 μm sections, mounted on glass slides, dewaxed, rehydrated through graded ethanols, and H&E-stained. Analyses were performed using a light microscope (Olympus, Japan). Skin samples were fixed with 10% neutralized formalin, embedded in paraffin, and used for immunohistochemistry or immunofluorescence staining using a rabbit polyclonal antibody as described previously.

Statistical study

Experiments were run independently in triplicate. Data are presented as means ± standard deviation (or standard error of values) obtained from the experiments. The student’s t-test was usually used to determine if two sets of data (follow normal distributions) were significantly different from each other. If the data did not follow a normal distribution, we should perform a non-parameter method, such as a Mann-Whitney U test, instead of a student’s t-test. If there were more than two sets of data which follow normal distributions, one-way ANOVA (one-way analysis of variance) was commonly used to determine whether the data sets were different from each other. The Student’s t-test was performed in figure 1b-1i, 2a-2b, 2f-2i, 3a-3b, 3e-3j, 4d-4j and 5a-5b, because there were comparisons between two different groups which followed normal distributions in triple duplicates. The non-parameter Mann-Whitney U test was utilized in figure 2c-2e, 3c-3d, 4a-4b and 5c-5g, because of the LC3 punctate or IHC positive points were not followed normal distributions. Furthermore, the one-way ANOVA was utilized in figure 1j and 1l to determine whether the data sets were different from each other.
